# Access to basic drinking water services, safe water storage, and household water treatment practice in rural communities of northwest Ethiopia

**DOI:** 10.1038/s41598-022-25001-y

**Published:** 2022-11-30

**Authors:** Zemichael Gizaw, Mulat Gebrehiwot, Bikes Destaw, Adane Nigusie

**Affiliations:** 1 Department of Environmental and Occupational Health and Safety, Institute of Public Health, College of Medicine and Health Sciences, University of Gondar, Gondar, Ethiopia; 2 Department of Health Education and Behavioral Sciences, Institute of Public Health, College of Medicine and Health Sciences, University of Gondar, Gondar, Ethiopia

**Keywords:** Environmental sciences, Risk factors

## Abstract

Protecting water from cross contamination at source and point of use is an important strategy to improve water quality. However, water safety measures at the source and point of use may not be implemented in the rural communities. This community-based cross-sectional study was, therefore, conducted among 1190 randomly selected households in a rural setting of northwest Ethiopia to assess access to basic drinking water services, safe water storage, and household water treatment practices. Water service level was determined using JMP criteria and practices that prevent cross contamination of water at point of use were used to determine safe water storage. Results showed that 23.0% of the households had access to basic water services; 37.0% practiced safe water storage; and 15.4% practiced one or more household water treatment methods. Public taps (54.5%) and protected spring (25.1%) were the common water sources to rural communities in northwest Ethiopia. Boiling (43.2%), chlorination or water guard (26.8%), and plain sedimentation (23.0%) were among the household water treatment methods commonly practiced in the area. In conclusion, rural households in the studied region has low access to basic water services. Safe water storage practice was also low in the area and household water treatment is not commonly practiced.

## Introduction

Lack of safe water remains one of the world’s most urgent health issues. People in developing countries have no access to safe and adequate drinking water despite access to safe drinking water is a global priority agenda. In 2015, it was estimated that 56% of the world's population had an unsafe water source^1^. Unsafe water sources are important sources of infectious diseases transmission^2–4^. The global burden of disease study estimated that in 2015, an unsafe water source resulted in 1.2 million deaths and 71.7 million disability-adjusted life years (DALYs)^1^. Access to safe drinking water together with hygiene and sanitation is fundamental to global health. Almost one tenth of the global disease burden could be prevented by increasing access to safe drinking water and improving sanitation and hygiene. Annually, safer water could prevent 1.4 million child deaths from diarrhea; 500,000 deaths from malaria; 860,000 child deaths from malnutrition; and 280,000 deaths from drowning. In addition, 5 million people can be protected from being seriously incapacitated from lymphatic filariasis and another 5 million from trachoma^5^.

Limited access to drinking water services continues to be a major public health problem in Ethiopia. In Ethiopia, provision of safe, accessible, and reliable water is very critical. In rural areas of Ethiopia during 2016, 4% have safely managed services, 30% have basic services, and 26% have limited services. This leaves 40% of the country’s rural population with unimproved water services^6^. According to Ministry of Water, Irrigation and Electricity (MoWIE) estimates, rural water supply coverage reached 63% by mid-2016^7^ (or 57% according to the Ethiopian Demographic and Health Survey 2016)^8^, but these assessments were not made based on the JMP service delivery categories.

Source-based water safety measures and protecting water from cross contamination at point of use are important strategies to improve water quality in the water supply system and to minimize associated health consequences. Source-based interventions include designing and construction of improved source that have the potential to protect water from contamination and deliver safe water; community-driven sanitation to protect pollution of the catchment area from human, animal, and agricultural wastes; and source-based water treatment^9^. Water safety measures at point of use include safe water storage and household water treatment^10–12^. However, these safety measures may not be implemented in the rural communities due to limited knowledge, misinformation, negative attitude, and lack of experience toward best practices of alternative water treatment technologies and safe storage^13^. Accordingly, this community-based cross-sectional study was conducted to assess access to basic drinking water services, safe water storage, and household water treatment practice in rural communities of northwest Ethiopia.

## Methods

### Study design and setting

**Figure 1:**
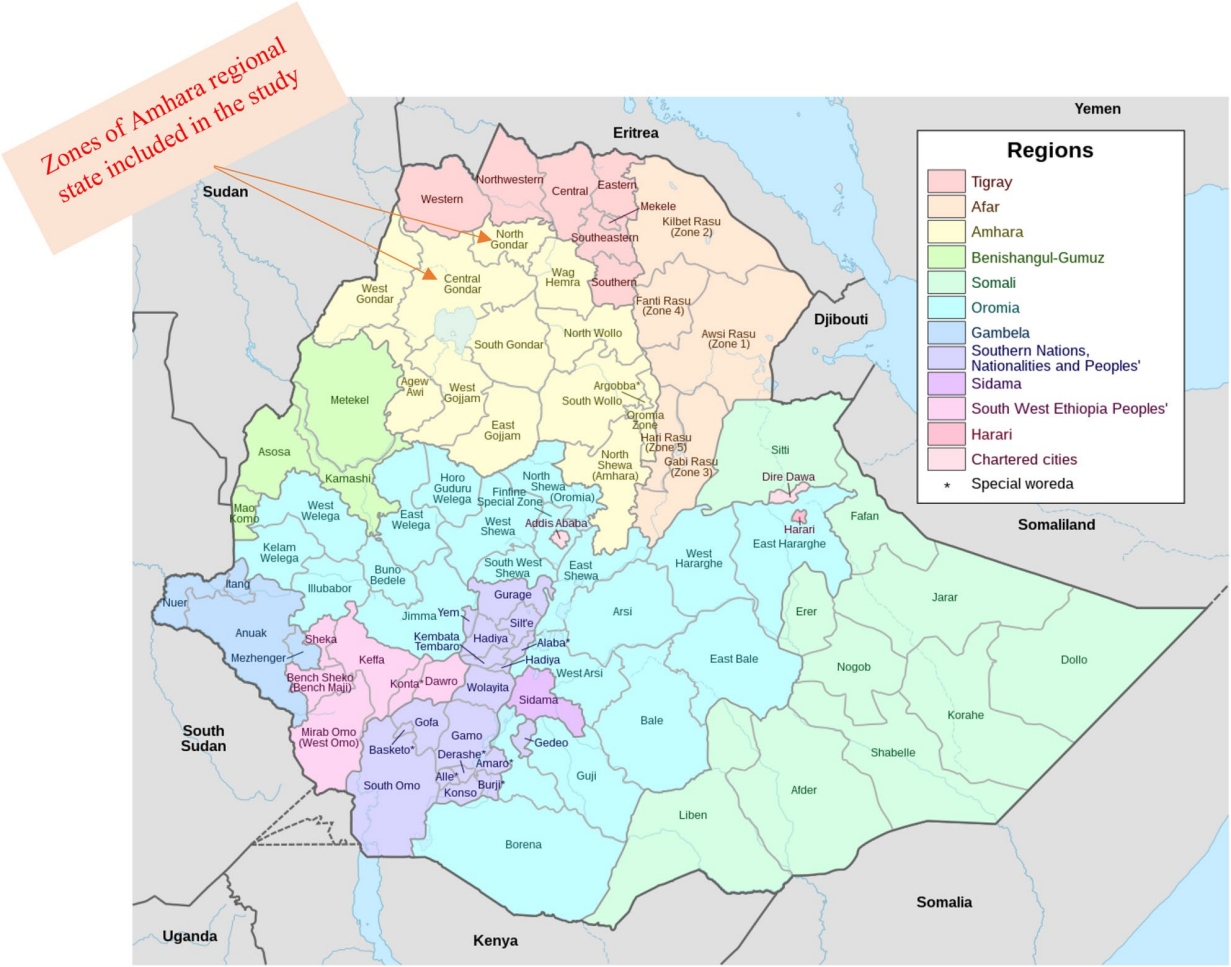
Map of study areas (Source: https://en.wikipedia.org/wiki/List_of_zones_of_Ethiopia#/media/File:Map_of_zones_of_Ethiopia.svg).

A community-based cross-sectional study with structured observation was conducted among rural households in Central and North Gondar administrative zones of the Amhara national regional state, Ethiopia in May 2016 (Fig. 1). Central Gondar zone covers thirteen districts and North Gondar zone covers seven districts. The total population residing in Central Gondar is estimated to be 2,896,928 and it is estimated to be 912,112 in North Gondar zone^14^.

### Sample size calculation and sampling procedures

The sample size (i.e., 1210 rural households) was calculated using single population proportion formula and the target households were included in the study using systematic random sampling technique. The sample size calculation and sampling procedures are described in more detail elsewhere^15^.

### Data collection tools and procedures

Structured and pretested questionnaire and spot-check observations were used to collect data. The questionnaire and observation checklists were prepared based on a review of relevant literature. The questionnaire was first prepared in English language and translated to the local Amharic language, and back-translated into English to check consistency. The questionnaire was organized in to three parts: (1) socio-demographic information; (2) access to WASH information; and (3) drinking water sources, handling practice, and household water treatment. Environmental health experts were participated in the data collection process after getting a one day training on the tool. The data collection process and completeness of data was closely supervised.

### Measurement of study variables

Access to basic drinking water services, safe water storage, and home-based water treatment were the primary outcomes of this study. Access to basic drinking water services was defined as drinking water from all year round improved source that have the potential to deliver safe water by nature of their design and construction, and include: piped water, public taps, protected wells, protected springs, and protected rain catchments, provided collection time is not more than 30 min for a roundtrip including queuing^16^. Safe water storage was defined as storing water in clean narrow-mouthed and properly covered containers plus withdrawing water from the storage containers by tilting or pouring^17^. Household water treatment is the application of different water treatment options including solar disinfection, chlorination (water guard), filtration, plain sedimentation, or boiling that improve water quality at the point of use^18^.

### Data processing and analysis

Data were entered using EPI-INFO version 3.5.3 statistical package and exported into Statistical Package for Social Sciences (SPSS) version 20 for further analysis. For most variables, data were presented by frequencies and percentages. We included predictors to the multivariable binary logistic regression model from the literature regardless of their bivariate p-value to identify factors associated with safe water storage and household water treatment. Statistically significant association was declared on the basis of adjusted odds ratio (AOR) with 95% confidence interval (CI) and p-values < 0.05. Model fitness was check using Hosmer and Lemeshow goodness-of-fit test.

### Ethics approval and consent to participate

Ethical clearance was obtained from the Institutional Review Board of the University of Gondar (reference number: V/P/RCS/05/1520/2016). There were no risks due to participation and the collected data were used only for this research purpose with complete confidentiality. Written informed consent was obtained from household heads. All the methods were carried out in accordance with relevant guidelines and regulations.

## Results

### Sociodemographic characteristics

A total of 1190 households were participated in the current study, with a response rate of 98.3%. The family size in 513 (43.1%) of the households was more than five and 1013 (85.1%) of the households had children. Three-forth, 888 (75.3%) and 643 (59.3%) of the female and male heads, respectively did not receive formal education. About half, 565 (47.5%) of the households reported that they received WASH education and 967 (81.3%) of the households reported that they have been regularly supervised by health professionals. Furthermore, 812 (68.2%) of the households reported that they regularly discussed about health and sanitation issues with their family. Similarly, 524 (44%) of the households reported that they discussed about health and sanitation issues with village health groups (Table 1).

**Table 1:** Sociodemographic characteristics of households (n = 1190) in a rural setting of northwest Ethiopia, May 2016.

Sociodemographic characteristics	Frequency	Percent
**Family size of households (n = 1190)**		
< 5	677	56.9
> 5	513	43.1
**The household has children**		
Yes	1013	85.1
No	177	14.9
**Maternal education (n = 1180)**		
No formal education	888	75.3
Attend formal education	292	24.7
**Paternal education (n = 1085)**		
No formal education	643	59.3
Attend formal education	442	40.7
**WASH education**		
Yes	565	47.5
No	625	52.5
**Health professionals’ regular supervision**		
Yes	967	81.3
No	223	18.7
**Discussion about WASH with the village health group?**		
No health group	287	24.1
Yes	524	44.0
No	379	31.8
**The family discussed about health issues**		
Yes	812	68.2
No	378	31.8

### Water sources

**Figure 2:**
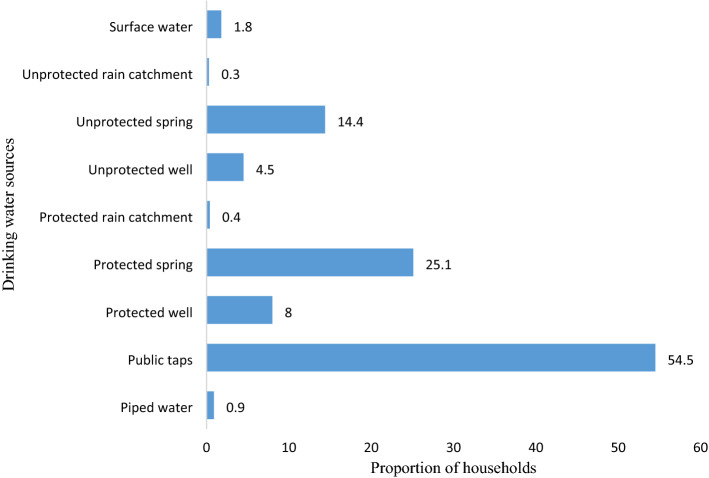
Drinking water sources for households (n = 1190) in a rural setting of northwest Ethiopia, May 2016.

In the current study, 957 (80.4%) of the households had access to improved water sources and more than half, 649 (54.5%) of the households collected drinking water from public taps (Fig. 2). The water sources for 156 (13.1%) households are not all year round and 837 (70.3%) of the households reported that the time for water collection is more than 30 min for a roundtrip including queuing time. The volume of water collected in 1154 (97.0%) of the households was below 20 L per capita per day. Accordingly, 274 (23.0%) (95% CI: 20.7, 25.3%) of the households had access to basic water services (Table 2).

**Table 2:** Access to basic water services among households (n = 1190) in a rural setting of northwest Ethiopia, May 2016.

Variables	Frequency	Percent
**Water sources**		
Unimproved	233	19.6
Improved	957	80.4
**Collection time for a roundtrip including queuing**		
$>$ 30 min	837	70.3
$\le$ 30 min	353	29.7
**Water sources are all year round**		
No	156	13.1
Yes	1034	86.9
**Volume of water collected**		
$<$ 20 l/c/d	1154	97.0
$\ge$ 20 l/c/d	36	3.0
**Access to basic waster services**		
Had no access to basic services	916	77.0
Had access to basic services	274	23.0

*l/c/d* liters per capita per day.

### Water handling at point of use

Two-third, 795 (66.8%) of the households primarily stored drinking water using narrow-mouthed containers, such as Jerricane. The water storage containers were clean at the time of the survey in 859 (72.2%) of the households and 544 (45.7%) of the households reported that they daily washed or cleaned the water storage containers. Moreover, the water storage containers were properly covered at the time of the survey in 1046 (87.9%) of the households and 768 (64.5%) of the households withdraw water from the storage containers by pouring or tilting. Accordingly, 440 (37.0%) (95% CI: 34.2, 39.6%) of the households practiced safe water storage (Table 3).

**Table 3:** Water handling practices of households (n = 1190) in a rural setting of northwest Ethiopia, May 2016.

Variables	Frequency	Percent
**Primary water storage containers**		
Wide-mouthed	395	33.2
Narrow-mouthed	795	66.8
**Water storage containers are clean**		
No	331	27.8
Yes	859	72.2
**How frequently households cleaned water storage containers**		
Daily	544	45.7
Every other day	115	9.7
Every two days	131	11.0
Every three days	139	11.7
Every four days	51	4.3
Every five days	35	2.9
Every six days	19	1.6
Every week	156	13.1
**Water storage containers are properly covered**		
No	144	12.1
Yes	1046	87.9
**Methods to withdraw water from the storage containers**		
Dipping	422	35.5
Tilting or pouring	768	64.5
**Safe water storage**		
No	750	63.0
Yes	440	37.0

### Household water treatment

The current study revealed that 183 (15.4%) (95% CI: 13.3, 17.5%) of the households practiced one or more household water treatment methods. Boiling [79 (43.2%)], chlorination or water guard [49 (26.8%)], and plain sedimentation [42 (23.0%)] were among the household water treatment methods commonly practiced in the rural households. We also investigated the reasons why households did not treated water at household-level and found that 780 (77.5%) of the households reported that they did not practiced household water treatment methods since they believed that the water is safe and 232 (23.0%) of the households did not practiced household water treatment methods due to knowledge or awareness gap (Table 4).

**Table 4:** Household water treatment practices among households (n = 1190) in a rural setting of northwest Ethiopia, May 2016.

Variables	Frequency	Percent
**Home-based water treatment**		
No	1007	84.6
Yes	183	15.4
**Homebased water treatment methods (n = 183)**		
Solar disinfection	5	2.7
Water guard	49	26.8
Boiling	79	43.2
Cloth filtration	30	16.4
Plain sedimentation	42	23.0
**Reasons for not practicing home-based water treatment (n = 1007)**		
Believed that water is safe	780	77.5
Treatment options are expensive	54	5.4
Do not know about home-based water treatment methods	232	23.0
Treatment options are not available	65	6.5

### Factors associated with safe water storage and household water treatment

Health education, health supervision, family discussion, maternal education, paternal education, and family size were entered in to the multivariable model to identify factors associated with safe water storage. In the adjusted model, safe water storage was significantly associated with health education, health supervision, and family size. The odds of safe water storage was 1.73 times higher among households who received health education in three months prior to the survey compared with households who did not receive health education (AOR: 1.73, 95% CI 1.30, 2.30). Similarly, households who have been regularly supervised by health professionals had higher odds to safely stored water compared with their counterparts (AOR: 1.63 (1.13, 2.35). Small family sized households had also 1.30 times more odds to safely stored water compared with large family sized households (AOR: 1.30, 95% CI 1.01, 1.67) (Table 5).

**Table 5:** Factors associated with safe water storage practice among households (n = 1190) in a rural setting of northwest Ethiopia, May 2016.

Variables	Safe water storage		COR with 95% CI	AOR with 95% CI
	Yes	No		
**Health education**				
Yes	176	389	1.62 (1.27, 2.05)	1.73 (1.30, 2.30)***
No	264	361	1.0	1.0
**Health supervision**				
Yes	361	606	1.09 (0.80, 1.47)	1.63 (1.13, 2.35)**
No	79	144	1.0	1.0
**Family discussion**				
Yes	285	285	0.78 (0.61, 1.00)	0.86 (0.63, 1.18)
No	155	155	1.0	1.0
**Maternal education**				
No formal education	321	567	1.0	1.0
Attend formal education	117	175	1.18 (0.90, 1.55)	1.21 (0.89, 1.65)
**Paternal education**				
No formal education	240	403	1.0	1.0
Attend formal education	163	279	0.98 (0.76, 1.26)	0.97 (0.73, 1.28)
**Family size**				
$\le$ 5	264	413	1.22 (0.96, 1.55)	1.30 (1.01, 1.67)*
$>$ 5	176	337	1.0	1.0

*AOR* adjusted odds ratio, *CI* confidence interval, *COR* crude odds ratio.

*Statistically significant at p < 0.05, **statistically significant at p < 0.01, ***statistically significant at p < 0.001, Hosmer and Lemeshow test = 0.219.

Health education, health supervision, family discussion, maternal education, water sources, family size, and presence of children in the household were entered in to the multivariable model to identify factors associated with household water treatment. In the adjusted model, household water treatment was statistically associated with health professionals close supervision and family discussion on WASH issues. The odds of practicing household water treatment was 1.91 times higher among households who have been frequently supervised by health professionals compared with their counterparts (AOR: 1.91, 95% CI 1.05, 3.46). Similarly, the odds of practicing household water treatment was 2.15 times higher among households who regularly discussed about WASH issues with their families compared with households who did not regularly discussed about WASH (AOR: 2.15, 95% CI 1.35, 3.43) (Table 6).

**Table 6:** Factors associated with household water treatment practice among households (n = 1190) in a rural setting of northwest Ethiopia, May 2016.

Variables	Household water treatment		COR with 95% CI	AOR with 95% CI
	Yes	No		
**Health education**				
Yes	110	455	1.83 (1.33, 2.52)	1.18 (0.82, 1.69)
No	73	552	1.0	1.0
**Health supervision**				
Yes	168	799	2.92 (1.68, 5.05)	1.91 (1.05, 3.46)*
No	15	208	1.0	1.0
**Family discussion**				
Yes	154	658	2.82 (1.86, 4.28)	2.15 (1.35, 3.43)***
No	29	349	1.0	1.0
**Maternal education**				
No formal education	130	758	1.0	1.0
Attend formal education	51	241	1.23 (0.87, 1.76)	1.22 (0.85, 1.75)
**Water sources**				
Unimproved	33	200	0.89 (0.59, 1.33)	1.02 (0.67, 1.55)
Improved	150	807	1.0	1.0
**Family size**				
$\le$ 5	104	573	1.00 (0.73, 1.37)	1.07 (0.77, 1.49)
$>$ 5	79	434	1.0	1.0
**The household has children**				
Yes	163	850	1.51 (0.92, 2.47)	1.40 (0.83, 2.37)
No	20	157	1.0	1.0

*AOR* adjusted odds ratio, *CI* confidence interval, *COR* crude odds ratio.

*Statistically significant at p < 0.05, *** statistically significant at p < 0.001, Hosmer and Lemeshow test = 0.412.

## Discussion

This is a community-based cross-sectional study conducted to assess access to basic water services, safe water storage, and household water treatment practice among households in a rural setting of northwest Ethiopia. This study found that 23.0% (95% CI 20.7, 25.3%) of the households had access to basic water services; 37.0% (95% CI 34.2, 39.6%) of the households practiced safe water storage; and 15.4% (95% CI 13.3, 17.5%) of the households practiced one or more household water treatment methods. Boiling (43.2%), chlorination or water guard (26.8%), and plain sedimentation (23.0%) were among the household water treatment methods commonly practiced in the rural households.

The proportion of households who had access to basic water services in the current study (i.e., 23%) is comparable with a report of JMP, i.e., 30% of households in rural areas of Ethiopia have basic services^6^. However, the proportion of households with basic services in the current study is lower than reports of the Ministry of Water, Irrigation and Electricity (MoWIE) and the 2016 Ethiopian Demographic and Health Survey. Ministry of Water, Irrigation and Electricity reported that rural water supply coverage reached 63% by mid-2016^7^ and 57% according to the Ethiopian Demographic and Health Survey 2016)^8^. This differences might be due to the assessment methods used. In our study, we used the JMP definition to basic services as elaborated in the method part, whereas assessments in the aforementioned reports were not made based on the JMP service delivery categories, i.e., they only considered improved sources, which overestimates the coverage. Moreover, the lower access level to basic services in the current study might be due to the resilience of water sources in dry season since we collected data in the dry season. Most water sources in dry season are unreliable, which leads the community to use unimproved water sources in long distances^6^.

The proportion of households who safely stored water in the current study (i.e., 37%) is comparable with findings of a study in rural households of Oshimili North Local Government Area of Delta State, Nigeria, 40%^19^. On the other hand, the proportion of households who safely stored water in the studied region is lower than findings of studies in three districts of Amhara region, 58.8%^17^ and Bona District of Sidama zone, 78.1%^20^. This low-level safe storage practice in the study area might be due to the fact that water storage is affected by traditions such as use of wide-mouthed clay pots. Rural communities preferred to store water in wide-mouthed traditional clay pots because they believe that the use of a clay pot makes the water cool and thus “sweet to drink”^21,22^. However, the problem associated with this practice is the way they withdraw water, i.e., dipping of mugs, which largely cross contaminate the water. Moreover, rural communities may not have access to detergents to wash water storage containers due to poor socioeconomic status^23–25^ that makes the storage containers unsafe.

The proportion of households who practiced household water treatment in the current study (i.e., 15.4%) is comparable with findings of studies in Degadamot Woreda, northwest Ethiopia, 14%^26^ and Assosa Woreda of Benishangul Gumuz Region, 13.2%^27^. On the other hand, it is lower than findings of studies in Southern Ethiopia, 29.9%^28^; Ameya district of Oromia region, 30.3%^29^; Gibe District of Southern Ethiopia, 34.3%^23^; India, 53%^30^; Zambia, 50%^31^; Nigeria, 54%^32^; and Uganda, 76%^33^. The low-level practice of household water treatment in the studied region might be due to knowledge or awareness gap, perceived quality of drinking water, unavailability of treatment options, and cost. As documented in the current study, rural households did not practice household water treatment because of the following reasons: believing that the water is safe (77.5%), knowledge or awareness gap (23.0%), unavailability of treatment options (6.5%), and treatment options are expensive (5.4%). Over all, the low-level practice of household water treatment in the area can be due to psychological factors. Attitude towards water-related technology or behavior is the most important psychological factor to make people treat the drinking water^34–36^.

This study revealed that safe water storage was significantly associated with health education, health supervision, and family size. The odds of safe water storage was higher among households who received health education, who have been regularly supervised by health professionals, and who had small family size. Similarly, household water treatment was associated with health supervision and family discussion. The odds of practicing household water treatment was higher among households who have been frequently supervised by health professionals and among households who regularly discussed about WASH issues with their families. The effect of health education can be justified as health education encourages changes in healthy behaviors and it is an effective strategy to create demand for water safety measures and thereby increase good practice^35,37–39^. Moreover, health supervision is effective in improving or maintaining households’ WASH practices. Health supervision is critical in area where there is no other sources of health information and low self-determination to improve WASH^40^. The effect of large family size can be justified that large family number diverts attention of household heads to routine family supports than investing in water safety measures^41,42^. Moreover, large family sized households may have economic constrains and so that households may not have opportunities to invest on water safety measures^41,43^.

Lastly, to increase the degree to which inferences from the sample households can be generalized to a larger group of population (i.e., population validity), we recruited households at random or in a manner in which they are representative of the population that we wish to study and we granted that every household had an equal chance to be included in the study. In addition, we calculated adequately powered sample size using sample size determination procedures appropriate to objective with appropriate assumptions. Furthermore, our findings could be applicable to other situations and settings which have similar characteristics with the study populations of the current study, such as rural settings in developing countries (i.e., ecological validity). As limitations, the self-reported data may not be reliable since the study subjects may make the more socially acceptable answers rather than being truthful and they may not be able to assess themselves accurately, which might result reporting bias. Moreover, variables we included in the current study to identify factors associated with water handling/management practices are not complete.

## Conclusion

Rural households in the studied region has low access to basic water services. This low access to basic water services implies that the community is collecting water from unimproved water sources that results contamination of water with disease causing pathogens and chemicals at the source. Moreover, the proportion of households who safely stored water is low in the area, which may intensify the level of water contamination in the water supply chain. Furthermore, household water treatment is not commonly practiced in the study area that indicates protection of water sources from contamination and source-based water treatment are effective approaches to improve drinking water safety in the area. All these imply that access to safe water in a rural setting of northwest Ethiopia is very critical and the spread of water-borne diseases in the community might be high. The local health department in collaboration with the community and other stakeholders need, therefore, strongly work to design and construct communal water sources that have the potential to deliver safe and adequate water all year rounds. Moreover, maintaining the constructed water sources is important since most of the water infrastructures were damaged. In addition, promotion of water safety measures at point of use, such as safe water storage and household water treatment through health education, health supervision, and village discussions is critical.

## Data Availability

The datasets generated during and/or analyzed during the current study are available from the corresponding author on reasonable request.
